# Depression Screening and Rates of Mood Disorder Diagnosis and Suicide in Younger and Older Adults

**DOI:** 10.1093/geroni/igaa057.1188

**Published:** 2020-12-16

**Authors:** Julie Lutz, Kenneth Conner, Michael Hasselberg, Kathleen Fear, Kim Van Orden

**Affiliations:** 1 University of Rochester Medical Center, Rochester, New York, United States; 2 University of Rochester School of Medicine and Dentistry, Rochester, New York, United States

## Abstract

Rates of suicide are elevated among older adults in the U.S. and around the world, with the highest rates in older men (CDC, 2019; WHO, 2014). More than half of older adults who die by suicide were in contact with a primary care physician within the month prior to death, and almost one-third within the prior week (Luoma et al., 2002; Stene-Larsen & Reneflot, 2019), demonstrating the importance of better identification and treatment of mental health issues among older adults across healthcare systems. Depression, a well-established risk factor for suicide, goes under-diagnosed and under-treated, especially among older adults (Bryant, 2010). The University of Rochester Medical Center has led integration of patient-reported outcomes assessment via large-scale implementation of PROMIS measures across multiple departments. We compared results of PROMIS depression screening from 1/1/2015 to 8/31/2019 with mood disorder diagnoses within the year prior and year following screening. Twenty-six percent (39491/154669) of adults under age 65 and 23% (11694/51702) of those age 65 and older screened positive for mild, moderate, or severe depression. Whereas 29.0% of younger adults who screened positive received mood disorder diagnoses, 22.1% of older adults received a diagnosis (χ2(1)=214.69, p<.001). Suicide outcomes using National Death Index data will also be reported. Results confirm depression is underdiagnosed (and, by extension, likely undertreated) at all ages, but show a distinct disparity for older adults, which may put them at greater risk for negative outcomes such as suicide.

